# P-1949. Candida spp. colonization in critically ill patients: Risk factors and outcomes

**DOI:** 10.1093/ofid/ofaf695.2117

**Published:** 2026-01-11

**Authors:** María A Pérez-Ardila, Luisannys I Lozada Pinto, Truc Cecilia Tran, Rodrigo P Baptista, Diana Panesso-botero, Marissa Schettino Intriago, Husna Malikzad, Shiva Murali, Andrea M Detranaltes, An Dinh, Kavindra Singh, Shubhra Singh, Asmita Ghosh, Roberta Higgins, Giselle Ortiz, Abigail A Amaya, Muhammad Virk, Kirsten Rydell, Mary North Jones, Rachel Atterstrom, Blake M Hanson, Samuel A Shelburne, Tor Savidge, David B Corry, J Christian Perez, Cesar A Arias, Max W Adelman

**Affiliations:** Houston Methodist Research Institute, Houston, TX; Houston Methodist Academic Institute, Houston, TX; Houston Methodist Research Institute, Houston, TX; Houston Methodist Research Institute, Houston, TX; Houston Methodist Hospital, Houston, TX; Houston Methodist Research Institute, Houston, TX; Houston Methodist Hospital, Houston, TX; Houston Methodist Research Institute and Weill Cornell Medical College, Houston, Texas; Houston Methodist Hospital, Houston, TX; Houston Methodist Research Institute, Houston, TX; Houston methodist research institute, Houston, Texas; Houston Methodist Hospital, Houston, TX; Houston Methodist Hospital, Houston, TX; Houston Methodist Hospital, Houston, TX; Houston Methodist Hospital, Houston, TX; Houston Methodist Hospital, Houston, TX; Houston Methodist The Woodlands Hospitals, conroe, Texas; Houston Methodist Hospital, Houston, TX; Houston Methodist Research Institute, Houston, TX; Houston Methodist Hospital, Houston, TX; The University of Texas Health Science Center, Houston, Texas; University of Texas MD Anderson Cancer Center, Houston, Texas; Baylor College of Medicine, Houston, Texas; Baylor College of Medicine, Houston, Texas; UTHealth Houston, Houston, Texas; Houston Methodist and Weill Cornell Medical College, Houston, TX; Houston Methodist Hospital, Houston, TX

## Abstract

**Background:**

*Candida* spp. are important pathogens among intensive care unit (ICU) patients, and colonization is the first step toward invasive infection. This study describes the epidemiology, risk factors, and colonization dynamics of *Candida* in ICU patients.

**Figure:**
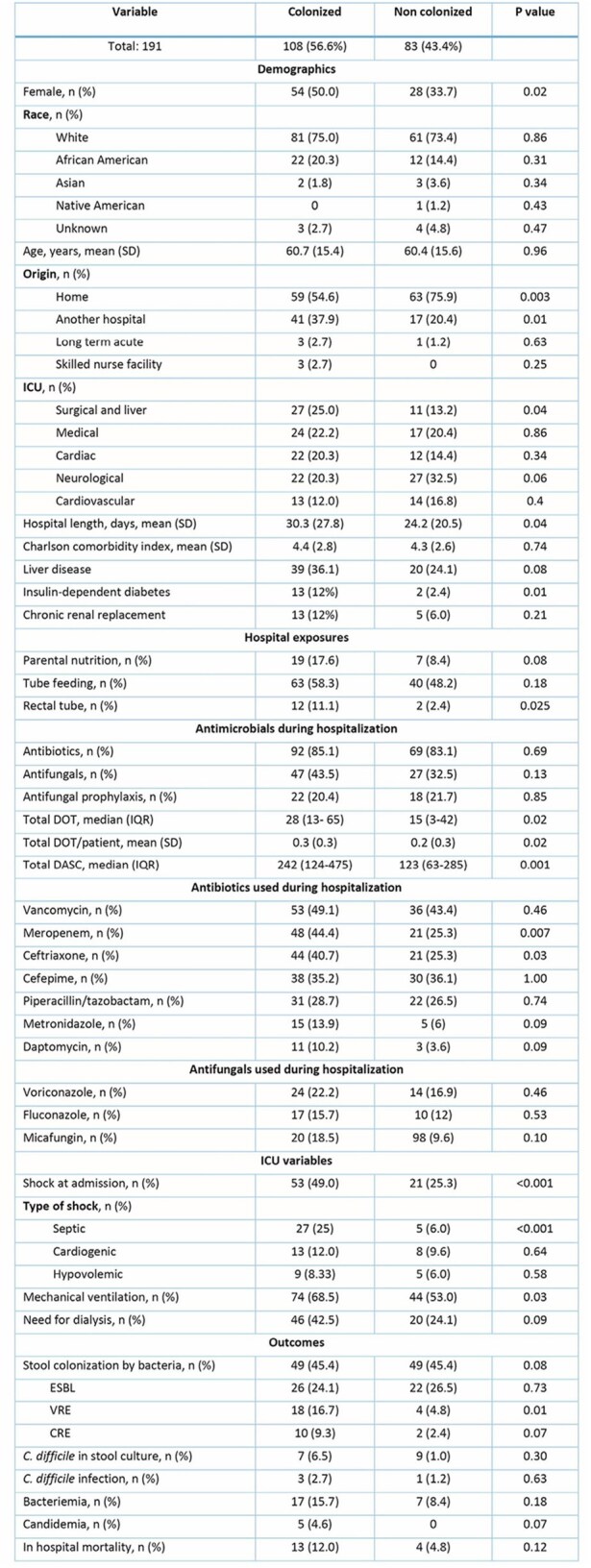

**Figure 1.**
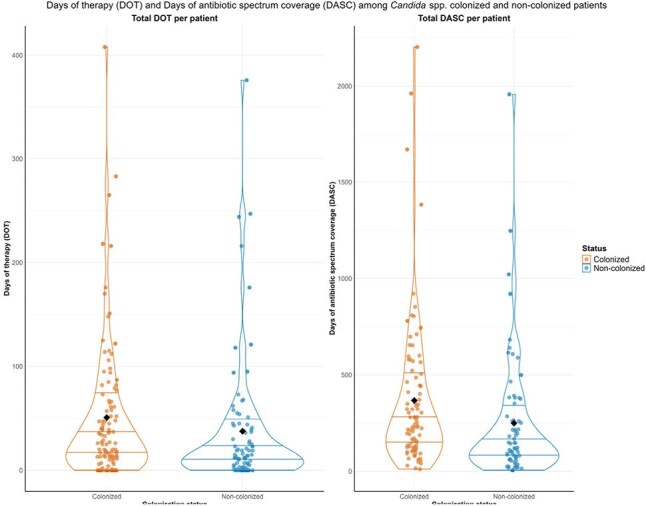
Days of therapy and days of antibiotic spectrum coverage among Candida spp. colonized and non-colonized patients.

**Methods:**

This nested case–control study included adult ICU patients who underwent fecal sample collection between 2021 and 2024. *Candida* colonization was detected via stool culture on *Candida* selective media and species were identified by MALDI-TOF. Patients were classified as either colonized or never colonized during ICU stay. Baseline characteristics, Total Days of Therapy (DOT), Days of Antibiotic Spectrum Coverage (DASC) and in-hospital mortality were compared. A time-dependent conditional logistic regression with a 1:2 matched design was used to identify risk factors for colonization, considering only exposures before the colonization day in cases and the matched day in controls.

**Figure 2.**
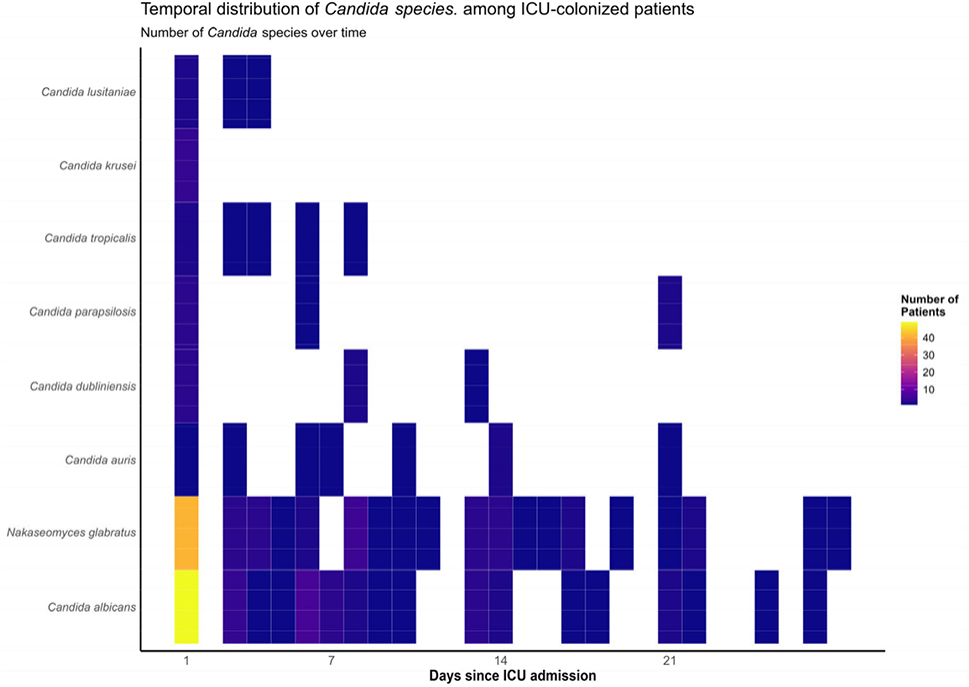
Temporal distribution of Candida species among ICU-colonized patients.

**Figure:**
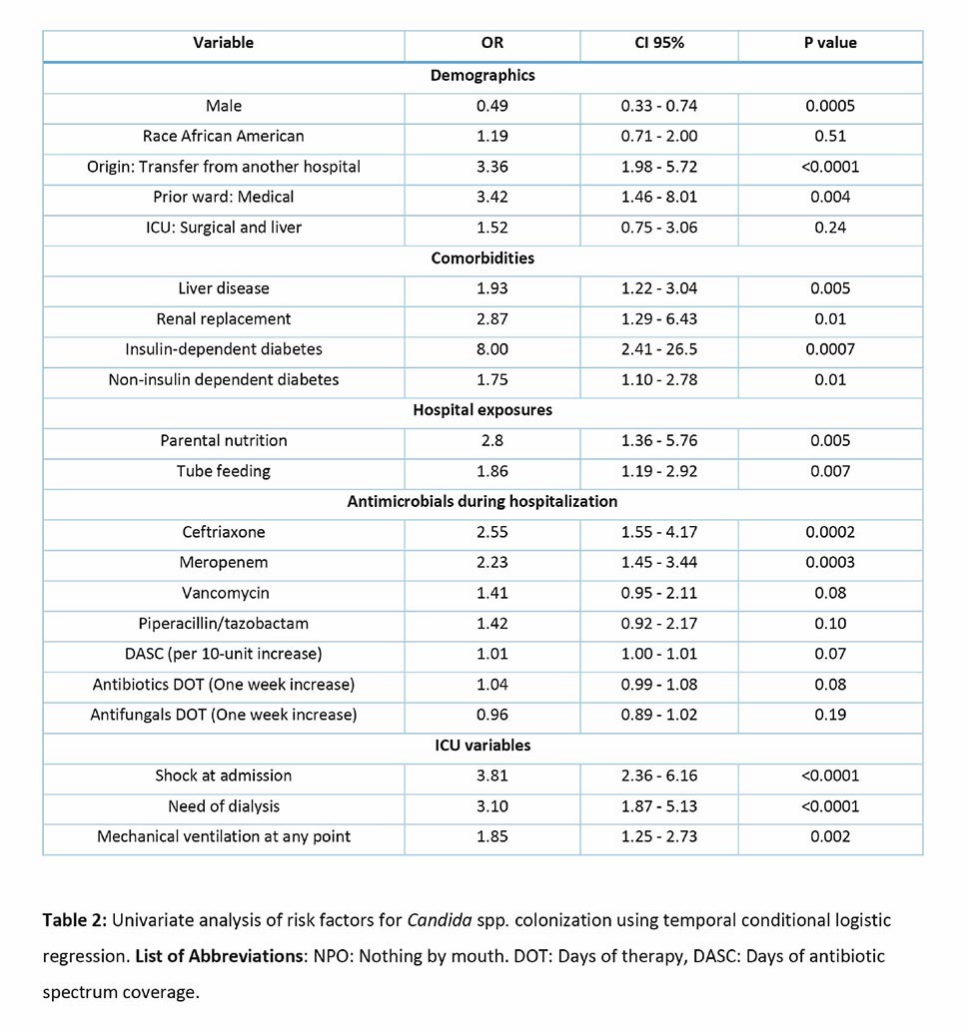

**Results:**

Among 191 ICU patients, 108 (56.6%) were colonized by *Candida* spp. Colonized patients had higher rates of liver disease and diabetes (Table 1). Colonized patients had greater exposure to vancomycin, meropenem, and ceftriaxone, along with higher DOT and DASC (Figure 1). In colonized patients, *C. albicans* was the most common species (52%), followed by *C. glabrata* (43%) (Figure 2). Co-colonization with other *Candida* species occurred in 14% of patients, and 25% experienced spontaneous clearance during their ICU stay. Colonized patients also had higher *Enterococcus* spp. colonization (24.1%). Univariate analysis showed that prior exposure to meropenem (odds ratio [OR] 2.2, 95% confidence interval [CI] 1.4-3.4) and ceftriaxone (OR 2.5, CI 1.5-4.1) were risk factors for colonization, while DOT and DASC were not significantly different between groups (Table 2). Candidemia occurred exclusively in colonized patients, although in cases (n=5) bloodstream species differed from those found in stool. Mortality did not differ by colonization status.

**Conclusion:**

*Candida* spp. colonization was common among ICU patients and often involved multiple species. Colonization was linked to increased broad antibiotic exposure. Colonized patients were more likely to have candidemia, although infecting species differed from the colonizing isolates.

**Disclosures:**

All Authors: No reported disclosures

